# End-of-Surgery Remimazolam Bolus Reduces Emergence Delirium in Children Undergoing Tonsillectomy and Adenoidectomy: A Randomized, Triple-Blind, Placebo-Controlled Trial

**DOI:** 10.3390/biomedicines14092115

**Published:** 2026-09-19

**Authors:** Grgur Prižmić, Filip Periš, Ana Maria Mitar, Ana Bego, Ana Šarić Jadrijev, Toni Kljaković-Gašpić

**Affiliations:** 1 Department of Anaesthesiology, Division of Anaesthesiology, University Hospital of Split, Spinčićeva 1, 21000 Split, Croatia; 2 School of Medicine, University of Split, Šoltanska 2, 21000 Split, Croatia; 3 Faculty of Health Sciences, University of Split, Ruđera Boškovića 35, 21000 Split, Croatia

**Keywords:** remimazolam, emergence delirium, pediatric anesthesia, sevoflurane, tonsillectomy, adenoidectomy, randomized controlled trial

## Abstract

**Background/Objectives**: Emergence delirium (ED) is common after sevoflurane anesthesia in children undergoing otolaryngologic surgery. Remimazolam is an ultra-short-acting benzodiazepine that may reduce ED without delaying recovery. This study evaluated the effect of a single remimazolam bolus administered at the end of surgery on ED in children undergoing tonsillectomy and/or adenoidectomy. **Methods**: In this prospective, randomized, triple-blind, placebo-controlled trial, 110 children aged 3–9 years with American Society of Anesthesiologists physical status I received remimazolam 0.1 mg/kg (*n* = 57) or an equal volume of normal saline (*n* = 53) immediately after discontinuation of sevoflurane and before extubation. The primary outcome was ED at extubation, defined as a Pediatric Anesthesia Emergence Delirium (PAED) score ≥ 10. Secondary outcomes included Face, Legs, Activity, Cry, and Consolability (FLACC) scores, emergence time, early recovery, postoperative vomiting, and airway complications. **Results**: ED occurred in 1 of 57 children (1.8%) receiving remimazolam and in 11 of 53 children (20.8%) receiving placebo (*p* = 0.002). The absolute risk reduction was 19.0% (95% confidence interval [CI], 7.5–31.8), the relative risk was 0.08 (95% CI, 0.01–0.63), and the number needed to treat was 6. The overall distribution of FLACC scores at extubation differed between groups (*p* = 0.047), with FLACC scores of 0 occurring more frequently in the remimazolam group. Median emergence time was 10 min in both groups, and all children achieved a modified Aldrete score > 8 within 15 min. Postoperative vomiting was assessed through 30 min; the comparison at 5 min showed no significant between-group difference. Airway complications also did not differ significantly between groups. **Conclusions**: A single intravenous bolus of remimazolam 0.1 mg/kg administered at the end of surgery significantly reduced PAED-defined ED without prolonging emergence or delaying early postoperative recovery.

## 1. Introduction

Emergence agitation (EA) and emergence delirium (ED) describe disturbed behavior during early recovery from general anesthesia. Although the terms are sometimes used interchangeably, ED refers more specifically to an acute disturbance of awareness, attention, and cognition, whereas EA is a broader behavioral construct that may result from pain, anxiety, discomfort, or disorientation. In the present study, ED was defined using the Pediatric Anesthesia Emergence Delirium (PAED) scale, a validated instrument developed specifically for the assessment of emergence behavior in children [1,2].

ED occurs predominantly in younger children and is particularly associated with volatile anesthetics such as sevoflurane. A recent systematic review identified younger age, preoperative anxiety, volatile anesthesia, rapid emergence, postoperative pain, and otolaryngologic or ophthalmic surgery as established or probable risk factors [3]. A 2024 systematic review and meta-analysis reported a pooled ED prevalence of approximately 19% and identified head and neck surgery as an important risk factor [4]. The reported incidence nevertheless varies widely because of differences in patient age, surgical setting, anesthetic technique, assessment instrument, diagnostic threshold, and timing of evaluation [2,3,4].

Although ED is generally self-limiting, its clinical consequences may be relevant. Affected children may cry inconsolably, thrash, remove intravenous catheters, disrupt dressings, injure themselves, or traumatize the operative site. These episodes may require rescue medication, additional staff involvement, and prolonged monitoring in the post-anesthesia care unit (PACU) [2,3,4]. In children undergoing tonsillectomy and/or adenoidectomy, uncontrolled movements and distress are particularly undesirable because pain, airway obstruction, vomiting, and postoperative bleeding may coexist during early recovery.

A major methodological challenge is differentiating ED from postoperative pain. Both conditions may present with crying, facial tension, motor restlessness, and inconsolability. The PAED scale evaluates eye contact, awareness of the surroundings, purposeful actions, restlessness, and consolability [1]. In contrast, the Face, Legs, Activity, Cry, and Consolability (FLACC) scale was developed to assess behavioral manifestations of pain in young children [5]. Concurrent application of the PAED and FLACC scales does not eliminate diagnostic overlap but allows a more clinically defensible interpretation than either instrument alone.

The pathophysiology of pediatric ED has not been fully elucidated. Proposed mechanisms include developmental vulnerability, preoperative anxiety, nociceptive stimulation, rapid elimination of volatile anesthetics, and asynchronous recovery of cortical and subcortical networks during the transition to wakefulness [2,6]. Preventive strategies include both non-pharmacological interventions and pharmacological approaches involving propofol, α2-adrenergic agonists, opioids, ketamine, and other anesthetic adjuncts. An optimal intervention should attenuate clinically relevant ED during the brief emergence period without prolonging unconsciousness or delaying postoperative recovery.

Remimazolam is an ultra-short-acting benzodiazepine that potentiates γ-aminobutyric acid type A receptor activity. It is rapidly hydrolyzed by tissue esterases to an inactive metabolite, resulting in a rapid onset, predictable offset, and limited accumulation [7]. Its effects can also be antagonized with flumazenil. These pharmacological characteristics may be advantageous during the transition from anesthesia to wakefulness, although pediatric experience remains more limited than that in adults [8].

In a randomized controlled trial involving children undergoing tonsillectomy and adenoidectomy under sevoflurane anesthesia, Yang et al. reported that remimazolam administered at the end of surgery significantly reduced the incidence of PAED-defined ED compared with saline [9]. An end-of-surgery remimazolam bolus subsequently reduced clinically relevant emergence agitation in adults undergoing rhinologic surgery without delaying early recovery [10]. However, additional evidence is required to determine whether a lower dose of remimazolam, 0.1 mg/kg, is effective in children undergoing tonsillectomy, adenoidectomy, or combined adenotonsillectomy.

The objective of this study was therefore to determine whether a single intravenous bolus of remimazolam 0.1 mg/kg administered immediately after discontinuation of sevoflurane and before extubation reduces the incidence of PAED-defined ED at extubation in children undergoing tonsillectomy and/or adenoidectomy.

## 2. Materials and Methods

### 2.1. Study Design, Ethics, and Registration

This prospective, randomized, triple-blind, placebo-controlled trial was conducted at the Department of Anaesthesiology, University Hospital of Split, Split, Croatia. The study protocol was approved by the Ethics Committee of University Hospital of Split before participant enrollment (approval number: 520-03/24-01/13; approval date: 29 January 2024). The trial was retrospectively registered on ClinicalTrials.gov (NCT07704437) on 7 July 2026 and was first posted on 15 July 2026 due to an administrative oversight. Written informed consent was obtained from a parent or legal guardian of each child before enrollment. The study was conducted in accordance with the Declaration of Helsinki and reported in accordance with the Consolidated Standards of Reporting Trials (CONSORT) guidelines.

### 2.2. Participants

Children aged 3–12 years with American Society of Anesthesiologists (ASA) physical status I who were scheduled for elective tonsillectomy, adenoidectomy, or combined adenotonsillectomy under general anesthesia were eligible for inclusion. Although the prespecified eligibility criterion included children aged 3–12 years, no eligible children aged 10–12 years presented during the recruitment period; consequently, the age range of the enrolled population was 3–9 years. All procedures were performed for established clinical indications in accordance with current international recommendations for tonsillectomy and/or adenoidectomy in children. The exclusion criteria were ASA physical status II or higher and known hypersensitivity to remimazolam or other benzodiazepines.

**Figure 1:**
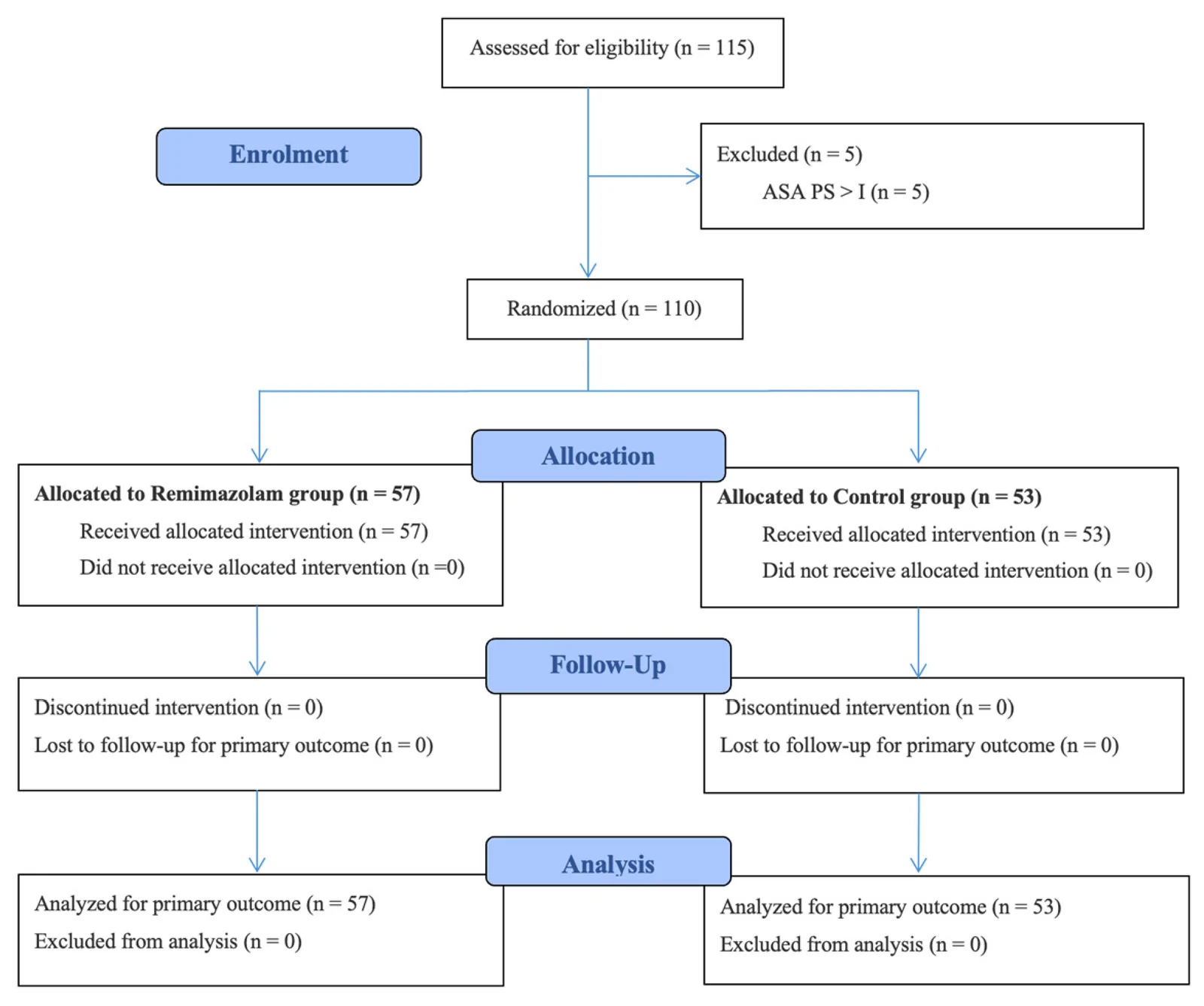
Consolidated Standards of Reporting Trials (CONSORT) diagram. ASA PS: American Society of Anesthesiologists physical status. Bold text indicates the allocated study groups.

Between April 2024 and July 2025, 115 children were assessed for eligibility. Five children were excluded because they had ASA physical status II or higher. A total of 110 children aged 3–9 years were enrolled and randomized to receive remimazolam (*n* = 57) or placebo (*n* = 53). All randomized participants received the allocated intervention, completed the study, and were included in the final analysis. No participants were lost to follow-up (Figure 1).

### 2.3. Randomization, Allocation Concealment, and Blinding

Participants were assigned to the remimazolam or placebo group using a computer-generated simple randomization sequence with an intended 1:1 allocation ratio. The sequence was applied consecutively according to the order of enrollment and was generated by an investigator who was not involved in participant recruitment, intraoperative management, study-drug administration, postoperative outcome assessment, data management, or statistical analysis.

Allocation concealment was maintained by an anesthesiologist who was not otherwise involved in the trial. This anesthesiologist prepared either remimazolam or normal saline in identical syringes containing equal volumes. The syringes were labeled only with the corresponding study identification number and were subsequently provided to the blinded treating anesthesiologist responsible for study-drug administration.

Remimazolam and normal saline are both clear, colorless solutions. No perceptible differences in appearance, viscosity, or syringe-plunger resistance were reported by the blinded anesthesiologist who administered the study medication.

Participants, treating anesthesiologists, the PACU outcome assessor, and the investigator performing the statistical analysis remained blinded to group allocation until completion of the analysis. The anesthesiologist who prepared the study medication was aware of group allocation but had no role in participant recruitment, perioperative management, study-drug administration, extubation, postoperative outcome assessment, data management, or statistical analysis. Blinding integrity was not formally assessed by asking the blinded anesthesiologists or outcome assessors to guess treatment allocation.

### 2.4. Anesthesia Protocol and Intervention

All participants received a standardized anesthetic protocol. No sedative premedication was administered. Anesthesia was induced with sevoflurane in oxygen until sufficient anesthetic depth was achieved to allow peripheral intravenous cannulation. After intravenous access had been secured, fentanyl 2 μg/kg and propofol 3 mg/kg were administered to facilitate tracheal intubation. Tracheal intubation was performed without the use of a neuromuscular blocking agent.

Anesthesia was maintained with sevoflurane in an oxygen–air mixture, titrated to maintain a bispectral index between 40 and 60. Mechanical ventilation was volume-controlled using a tidal volume of 6–8 mL/kg. The respiratory rate was adjusted to maintain an end-tidal carbon dioxide concentration between 35 and 40 mmHg. Before the start of surgery, an additional intravenous dose of fentanyl was administered according to the surgical procedure: 1 μg/kg for adenoidectomy, 2 μg/kg for tonsillectomy, and 3 μg/kg for combined tonsillectomy and adenoidectomy.

At the beginning of surgery, all participants received dexamethasone 0.1 mg/kg intravenously. Shortly before the end of surgery, paracetamol 15 mg/kg and granisetron 0.02 mg/kg were administered intravenously.

At the end of surgery, sevoflurane was discontinued. Immediately thereafter and before extubation, participants received either remimazolam 0.1 mg/kg or an equal volume of normal saline as a single intravenous bolus. Awake tracheal extubation was performed after the return of adequate spontaneous ventilation, purposeful responsiveness, and protective airway reflexes.

After completion of the initial PAED and FLACC assessments, rescue sedation with intravenous midazolam 0.05 mg/kg was administered to children with a PAED score ≥ 10. Rescue analgesia with intravenous fentanyl 1 μg/kg was administered to children with a FLACC score ≥ 4.

### 2.5. Outcomes

Baseline demographic and clinical variables potentially relevant to the occurrence of emergence delirium included age, sex, height, weight, body mass index, and type of surgical procedure. The total intraoperative fentanyl dose administered before emergence was also recorded.

Emergence delirium was assessed using the Pediatric Anesthesia Emergence Delirium (PAED) scale. The PAED scale consists of five items, each scored from 0 to 4, yielding a total score ranging from 0 to 20. A PAED score ≥ 10 was prespecified in the study protocol as the threshold for PAED-defined emergence delirium. Because no single PAED cutoff is universally accepted, the robustness of the primary finding was additionally explored in a post hoc sensitivity analysis using a more stringent threshold of PAED ≥ 12. The initial PAED assessment was performed in the operating room immediately after extubation and before the administration of any rescue medication.

PAED and FLACC assessments were repeated 10, 20, and 30 min after extubation for descriptive clinical monitoring. The prespecified primary efficacy analysis was based on the initial assessment performed immediately after extubation. Pain-related behavior was assessed using the Face, Legs, Activity, Cry, and Consolability (FLACC) scale concurrently with the PAED assessment at each predefined time point to facilitate differentiation between pain-related behavior and emergence delirium. The FLACC scale ranges from 0 to 10, with higher scores indicating more pronounced pain-related behavior.

Emergence time was defined as the interval between discontinuation of sevoflurane and tracheal extubation. Early postoperative recovery was assessed using the modified Aldrete score at 5-min intervals after extubation. Achievement of a modified Aldrete score > 8 was considered fulfillment of the prespecified early recovery criterion.

Postoperative vomiting (POV) was assessed by the blinded PACU outcome assessor at 5, 10, 20, and 30 min after extubation and recorded as a binary outcome (present or absent) at each time point. POV was recorded before the administration of rescue antiemetic treatment. All children who experienced POV received intravenous metoclopramide 0.1 mg/kg. Following each episode of vomiting, the surgical site was inspected to exclude immediate postoperative hemorrhage. Airway complications included laryngospasm and bronchospasm. Bronchospasm was defined as newly developed expiratory wheezing accompanied by prolonged expiration and/or increased respiratory effort. The occurrence and treatment of airway complications were recorded.

The administration of rescue sedation, rescue analgesia, and rescue antiemetic treatment was recorded.

### 2.6. Sample Size

The final sample-size requirement was determined following a blinded internal pilot analysis of the first 20 enrolled participants (10 per coded group) using G*Power version 3.1.9.7 (Heinrich Heine University Düsseldorf, Düsseldorf, Germany). The investigator performing the internal pilot analysis and sample-size calculation remained blinded to treatment allocation, with the two groups identified only by coded labels. In the internal pilot, PAED-defined emergence delirium occurred in 2 of 10 children (20%) in one coded group and in 0 of 10 children (0%) in the other. Because assuming a zero event rate could overestimate the anticipated treatment effect, an incidence of 2% was used for the lower-incidence group in the formal sample-size calculation, while the observed 20% incidence in the other group was retained. These anticipated incidences corresponded to a Cohen’s h effect size of approximately 0.64. With 80% statistical power and a two-sided α of 0.05, the minimum required total sample size was 96 participants. To account for potential attrition, the target sample size was increased to 106 participants. Because the prespecified randomization sequence contained 110 allocations, recruitment continued until 110 children had been enrolled. Participants included in the internal pilot remained in the final intention-to-treat analysis.

### 2.7. Statistical Analysis

Statistical analyses were performed using MedCalc Statistical Software version 22.014 (MedCalc Software Ltd., Ostend, Belgium). The distribution of continuous variables was assessed using the Shapiro–Wilk test.

Normally distributed continuous variables were presented as mean ± standard deviation and compared using the independent-samples *t*-test. Non-normally distributed continuous variables were presented as median and first and third quartiles and compared using the Mann–Whitney U test. Categorical variables were presented as numbers and percentages and compared using Pearson’s chi-square test or Fisher’s exact test, as appropriate. The distribution of FLACC categories (0, 1–3, and ≥4) between groups was compared using the Fisher–Freeman–Halton exact test.

For the primary outcome, relative risk and absolute risk reduction were calculated with corresponding 95% confidence intervals. The number needed to treat was calculated as the reciprocal of the absolute risk reduction. All statistical tests were two-sided, and *p* < 0.05 was considered statistically significant. All randomized participants were analyzed according to their assigned study group.

As a post hoc sensitivity analysis, the association between treatment allocation and ED was evaluated after stratification by surgical procedure (adenoidectomy, tonsillectomy, or combined tonsillectomy and adenoidectomy) using the Mantel–Haenszel method. The common odds ratio (OR) with 95% CI was calculated, and homogeneity of the treatment effect across surgical strata was assessed using the Breslow–Day test. As a post hoc sensitivity analysis, the primary outcome was re-evaluated using a more stringent PAED threshold of ≥12, with between-group proportions compared using Fisher’s exact test.

## 3. Results

A total of 110 children aged 3–9 years were randomized and included in the final analysis: 57 in the remimazolam group and 53 in the control group. All randomized participants received the allocated intervention, and no participants were lost to follow-up or excluded from the analysis (Figure 1). Baseline demographic and clinical characteristics are summarized in Table 1.

The distribution of surgical procedures is presented in Table 2.

The median total intraoperative fentanyl dose administered before emergence was 4.0 (3.0, 5.0) μg/kg in the control group and 4.0 (3.0, 5.0) μg/kg in the remimazolam group (*p* = 0.389, Mann–Whitney U test).

### 3.1. Emergence Delirium

PAED-defined ED at extubation was significantly less frequent in the remimazolam group than in the control group (1.8% vs. 20.8%; *p* = 0.002). The absolute risk reduction was 19.0 percentage points (95% CI, 7.5–31.8), the relative risk was 0.08 (95% CI, 0.01–0.63), and the number needed to treat was 6. In a post hoc sensitivity analysis using a more stringent PAED threshold of ≥12, ED occurred in 4 of 53 children (7.5%) in the control group and in 0 of 57 children (0%) in the remimazolam group (Fisher’s exact test, *p* = 0.051).

No child with a PAED score < 10 immediately after extubation subsequently developed a PAED score ≥ 10 at 10, 20, or 30 min after extubation.

PAED scores at 10, 20, and 30 min after extubation are presented descriptively in Supplementary Table S1 to illustrate their longitudinal course during the early postoperative period.

### 3.2. Emergence Delirium According to Surgical Procedure

ED occurred in 1 of 17 (5.9%) versus 5 of 18 (27.8%) children undergoing adenoidectomy, 0 of 13 (0%) versus 3 of 15 (20.0%) undergoing tonsillectomy, and 0 of 27 (0%) versus 3 of 20 (15.0%) undergoing combined tonsillectomy and adenoidectomy in the remimazolam and control groups, respectively. After adjustment for surgical procedure, the association between remimazolam and lower odds of ED remained significant (Mantel–Haenszel common OR, 0.07; 95% CI, 0.01–0.58; *p* = 0.002), with no evidence of heterogeneity across surgical procedures (Breslow–Day *p* = 0.568).

### 3.3. Pain-Related Behavior

The overall distribution of FLACC scores at extubation differed significantly between groups (Fisher–Freeman–Halton exact test, *p* = 0.047), with FLACC scores of 0 observed more frequently and FLACC scores ≥ 4 less frequently in the remimazolam group than in the control group (Table 3).

FLACC scores at 10, 20, and 30 min after extubation are also presented descriptively in Supplementary Table S1 to illustrate the longitudinal course of pain-related behavior.

### 3.4. Rescue Medications

Rescue midazolam was administered to 11 of 53 children (20.8%) in the control group and to 1 of 57 children (1.8%) in the remimazolam group. Rescue fentanyl was administered to 6 of 53 children (11.3%) in the control group and to 1 of 57 children (1.8%) in the remimazolam group. Five children in the control group and one child in the remimazolam group received both rescue midazolam and rescue fentanyl. All rescue medications were administered after completion of the initial PAED and FLACC assessments. All five children who experienced POV received intravenous metoclopramide 0.1 mg/kg.

### 3.5. Emergence Time and Early Recovery

Median emergence time was 10 (9, 13) min in the control group and 10 (10, 12) min in the remimazolam group, with no significant between-group difference (*p* = 0.666, Mann–Whitney U test). All children achieved a modified Aldrete score > 8 within 15 min after extubation.

### 3.6. Complications and Safety Outcomes

The inferential comparison of POV at 5 min after extubation showed no statistically significant between-group difference; observations at 10, 20, and 30 min were descriptive. No statistically significant between-group differences were observed in the recorded airway complications (Table 4). Postoperative vomiting occurred in 4 of 53 children (7.5%) in the control group and in 1 of 57 children (1.8%) in the remimazolam group (*p* = 0.194). For postoperative vomiting 5 min after extubation, the relative risk with remimazolam compared with placebo was 0.23 (95% CI, 0.03–2.01). At 10, 20, and 30 min after extubation, vomiting occurred in 2 (3.8%), 3 (5.7%), and 3 (5.7%) control-group children, respectively, compared with 1 (1.8%) child at each time point in the remimazolam group. Inspection of the surgical site revealed no evidence of immediate postoperative hemorrhage, and no child required immediate surgical re-exploration. No post-extubation laryngospasm occurred. Bronchospasm occurred in three children in the control group and in none in the remimazolam group. All bronchospasm episodes were mild and were treated with 100% oxygen and bronchodilators as clinically indicated.

## 4. Discussion

In this prospective, randomized, triple-blind, placebo-controlled trial, a single intravenous bolus of remimazolam 0.1 mg/kg administered immediately after discontinuation of sevoflurane and before extubation substantially reduced the incidence of PAED-defined ED at extubation in children undergoing tonsillectomy and/or adenoidectomy. ED occurred in 20.8% of children in the control group and in 1.8% of those receiving remimazolam, corresponding to an absolute risk reduction of 19.0 percentage points, a relative risk of 0.08, and a number needed to treat of six. In the post hoc sensitivity analysis using the more stringent PAED threshold of ≥12, the direction of the treatment effect was unchanged, although the between-group difference did not reach conventional statistical significance. The reduction in ED was not associated with delayed emergence: median emergence time was 10 min in both groups, and all children achieved a modified Aldrete score > 8 within 15 min. The absence of a detectable difference in emergence time and early Aldrete recovery argues against a clinically apparent delay in awakening, although subtle residual sedation cannot be excluded.

The present results are consistent with those of Yang et al., who reported that remimazolam 0.2 mg/kg administered at the end of surgery reduced the incidence of ED in children undergoing tonsillectomy and adenoidectomy under sevoflurane anesthesia [9]. The current trial demonstrates a similar direction of effect using a lower dose of 0.1 mg/kg. Differences in absolute event rates between the two studies should not be interpreted as evidence of the superiority of either dose. The incidence of pediatric ED is influenced by several factors, including age, preoperative anxiety, surgical procedure, perioperative analgesia, assessment timing, diagnostic threshold, and institutional recovery practices [2,3,4]. Consistency in the direction of the treatment effect is therefore more informative than direct numerical comparison of ED rates across studies.

Additional pediatric evidence supports the potential preventive effect of remimazolam. Cai et al. compared a continuous remimazolam infusion, a single remimazolam bolus administered near the end of surgery, and placebo in children undergoing laparoscopic surgery under sevoflurane anesthesia [11]. Both remimazolam regimens reduced ED compared with placebo. Lu et al. similarly reported a lower incidence of sevoflurane-associated ED following end-of-surgery remimazolam in children undergoing laparoscopic inguinal hernia repair [12]. Although these studies involved non-otolaryngologic procedures, their findings suggest that the potential effect of remimazolam may not be confined to adenotonsillar surgery. Direct comparison remains limited by differences in surgical stimulation, analgesic regimens, age distribution, timing of administration, remimazolam dose, and outcome definitions.

The present findings are also directionally consistent with our previous randomized adult trial, in which remimazolam 0.1 mg/kg administered after discontinuation of sevoflurane reduced clinically relevant emergence agitation following rhinologic surgery without delaying early recovery [10]. Adult emergence agitation assessed using the Richmond Agitation–Sedation Scale and pediatric ED assessed using the PAED scale are related but distinct clinical constructs. The adult study therefore supports the feasibility of an end-of-surgery bolus strategy but should not be regarded as direct evidence of pediatric efficacy.

The selected remimazolam dose was intended to attenuate ED without inducing renewed loss of consciousness or delaying recovery. Pediatric pharmacodynamic studies have reported age-dependent doses required for remimazolam-induced loss of consciousness that are substantially higher than the 0.1 mg/kg dose used in the present trial [13]. More directly relevant, Xue et al. estimated the 90% effective dose to be approximately 0.114 mg/kg for a single remimazolam bolus used to prevent ED following sevoflurane anesthesia [14]. The selected dose was therefore close to this estimate and may represent an appropriate balance between preventive efficacy and preservation of rapid emergence. However, dose estimates obtained from different populations, surgical procedures, and administration protocols should be compared cautiously.

Continuous-infusion data should be interpreted separately from single-bolus regimens. Yang et al. evaluated the effective dose of a continuous intraoperative remimazolam infusion for preventing ED in children undergoing adenoidectomy or adenotonsillectomy under sevoflurane anesthesia [15]. An infusion rate expressed in mg/kg/h cannot be directly extrapolated to a single bolus administered immediately before emergence. Nevertheless, these findings support the broader hypothesis that remimazolam exposure during sevoflurane anesthesia may reduce the occurrence of ED.

The mechanism underlying the observed reduction in ED remains uncertain. Pediatric ED probably results from an interaction among developmental susceptibility, preoperative anxiety, nociceptive stimulation, volatile anesthesia, and transiently disorganized restoration of awareness during emergence [2,3,4,6]. Remimazolam produces short-lasting modulation of γ-aminobutyric acid type A receptor activity and is rapidly hydrolyzed to an inactive metabolite [7,8]. Administration immediately before emergence may therefore attenuate excessive behavioral arousal during the transition from sevoflurane anesthesia to organized wakefulness. This explanation remains hypothetical because electroencephalographic activity and the sequence of cognitive recovery were not evaluated. Nevertheless, comparable emergence times and rapid achievement of the modified Aldrete threshold make prolonged unconsciousness an unlikely explanation for the lower incidence of PAED-defined ED.

The magnitude of the observed effect may be clinically relevant. A number needed to treat of six indicates that, under the conditions of the present trial, six children would need to receive remimazolam to prevent one episode of PAED-defined ED at extubation. This finding supports the potential usefulness of the intervention, particularly in children considered to be at increased risk of ED. However, the number needed to treat depends on the baseline incidence of ED and may differ according to age, surgical procedure, anesthetic technique, analgesic protocol, diagnostic threshold, and institutional practice.

The findings should also be considered in relation to other preventive strategies. Prophylactic dexmedetomidine has been shown to reduce the incidence of pediatric ED [16], while propofol-based strategies have also demonstrated preventive efficacy in children receiving sevoflurane anesthesia [17]. However, these agents differ in their pharmacodynamic, hemodynamic, respiratory, and recovery profiles. A potential practical advantage of remimazolam is that a single low-dose bolus can be incorporated into a conventional sevoflurane-based anesthetic without requiring a continuous infusion or conversion to total intravenous anesthesia. Because the present trial used saline as the comparator, it cannot establish whether remimazolam is superior, equivalent, or inferior to dexmedetomidine, propofol, or other active preventive interventions.

The FLACC findings require cautious interpretation. The overall distribution of FLACC categories differed between groups, with a greater proportion of children in the remimazolam group having a FLACC score of 0 and fewer having FLACC scores ≥ 4. Individual FLACC categories were not analyzed as separate confirmatory comparisons. Remimazolam has no established intrinsic analgesic effect, and lower FLACC scores should therefore not be interpreted as evidence of improved postoperative analgesia. Total intraoperative fentanyl exposure before emergence was comparable between groups, reducing the likelihood that the observed differences in FLACC scores were attributable to unequal opioid administration.

The PAED and FLACC scales share several observable behavioral components, including crying, motor activity, facial expression, and consolability [1,5]. Reduced behavioral arousal may therefore lower both scores without changing the underlying nociceptive input. Somaini et al. demonstrated that ED and pain may be better differentiated by individual behavioral characteristics than by total scores alone [18]. Lack of eye contact and reduced awareness of the surroundings were more characteristic of ED, whereas abnormal facial expression, crying, and inconsolability were more closely associated with pain. The most defensible interpretation of the present findings is therefore that the reduction in PAED-defined ED was not accompanied by an increase in pain-related behavior.

Early postoperative recovery was comparable between groups. Median emergence time was identical, and all children achieved a modified Aldrete score > 8 within 15 min after extubation. These findings argue against a clinically relevant delay in awakening after administration of remimazolam. However, the modified Aldrete score is a global recovery instrument and may not detect subtle residual sedation. Achievement of the predefined score also does not necessarily correspond to actual PACU discharge. Future studies should distinguish between time to eye opening, purposeful response, extubation, fulfillment of discharge criteria, and actual PACU discharge and should include a dedicated postoperative sedation scale.

The inferential comparison at 5 min showed no significant between-group difference in POV, and the subsequent observations through 30 min were descriptive. No significant differences were identified in laryngospasm or bronchospasm. Although all three episodes of bronchospasm occurred in the control group, the study was not powered to evaluate uncommon airway complications, and a protective effect of remimazolam cannot be inferred. Broader pediatric evidence provides supportive but indirect safety information. Kimoto et al. described the use of remimazolam as an adjunct to general anesthesia in a cohort of 418 children [19], while Fang et al. reported an acceptable efficacy and safety profile in a multicenter randomized comparison of remimazolam and propofol for pediatric general anesthesia [20]. However, the doses and administration strategies used in those studies differed substantially from the single 0.1 mg/kg emergence-phase bolus evaluated in the present trial.

The principal strengths of this study include its prospective randomized design, placebo control, comprehensive blinding of participants, care providers, the postoperative outcome assessor, and the statistical analyst, complete follow-up, and use of a standardized sevoflurane-based anesthetic protocol. The primary endpoint was clearly defined, and the treatment effect was reported using both absolute and relative measures. Concurrent PAED and FLACC assessment also addressed an important source of diagnostic uncertainty, although the behavioral overlap between ED and postoperative pain could not be eliminated.

Several limitations should be acknowledged. First, this was a single-center study that included only children with ASA physical status I, which limits generalizability to children with comorbidities, sleep-disordered breathing, complex airway disease, or different perioperative protocols. Second, preoperative anxiety, temperament, parental anxiety, previous anesthetic exposure, and behavior during mask induction were not assessed using validated instruments, despite their recognized association with ED [3,4]. Third, total PAED and FLACC scores cannot fully distinguish ED from pain, fear, anxiety, or normal postoperative distress, and item-level analyses were not performed. Exact end-tidal sevoflurane concentrations over time and cumulative exposure duration were not prospectively recorded; therefore, sevoflurane exposure could not be quantified or compared between groups. Moreover, POV was assessed only during the first 30 min after extubation; consequently, these findings should not be interpreted as the incidence of POV during the first 24 postoperative hours. A dedicated postoperative sedation scale was not included in the study protocol. Although emergence time and early modified Aldrete recovery were comparable between groups, and no child with an initial PAED score < 10 subsequently reached the threshold for PAED-defined emergence delirium during the 30-min observation period, subtle residual sedation as a potential contributor to lower PAED scores cannot be excluded. Because the sample-size assumptions were derived from the internal pilot, and these participants were retained in the final analysis, the close correspondence between the anticipated and observed event rates should be interpreted cautiously. The pilot and final estimates are not fully independent, and the internal pilot may therefore have contributed to an optimistic estimate of the treatment effect. Finally, the sample size was calculated for the primary efficacy endpoint and was insufficient to evaluate uncommon respiratory, airway, or other adverse events.

Further multicenter trials should compare remimazolam directly with active preventive interventions such as dexmedetomidine and propofol and should include children with a broader range of ages and perioperative risk. Dose-ranging studies may clarify whether the optimal remimazolam bolus differs according to age, surgical procedure, duration of sevoflurane exposure, or baseline risk of ED. Future studies should also incorporate serial and peak PAED scores, item-level PAED analyses, validated sedation assessment, standardized rescue medication criteria, actual PACU discharge time, and post-discharge behavioral outcomes.

## 5. Conclusions

In this single-center study, a single intravenous bolus of remimazolam 0.1 mg/kg administered immediately after discontinuation of sevoflurane and before extubation significantly reduced the incidence of PAED-defined emergence delirium at extubation in children undergoing tonsillectomy and/or adenoidectomy, without prolonging emergence time or early postoperative recovery.

## Figures and Tables

**Table 1:** Baseline and perioperative characteristics of the study population.

Variable	Control (*n* = 53)	Remimazolam (*n* = 57)	*p*-Value
Sex, M/F	24 (45.3)/29 (54.7)	28 (49.1)/29 (50.9)	0.687 *
Age (yr)	5.0 (4.0, 7.0)	5.0 (4.0, 6.0)	0.930 †
Height (cm)	111.3 ± 13.4	110.4 ± 14.0	0.736 ‡
Weight (kg)	18.0 (16.0, 21.0)	18.0 (16.0, 23.0)	0.692 †
BMI (kg/m^2^)	15.28 ± 1.92	15.90 ± 2.05	0.103 ‡
Operation duration (minutes)	40.0 (25.0, 48.0)	36.0 (27.0, 48.0)	0.834 †
Anesthesia duration (minutes)	65.0 (49.0–69.0)	57.0 (48.0–70.0)	0.943 †

Values are presented as mean ± standard deviation (SD) for normally distributed continuous variables, median (Q1, Q3) for non-normally distributed continuous variables, or number (%), as appropriate. M/F: male/female; BMI: body mass index; Q1 and Q3: first and third quartiles. * Pearson’s chi-square test; † Mann–Whitney U test; ‡ independent-samples *t*-test.

**Table 2:** Distribution of surgical procedures by treatment group.

Surgical Procedure	Control (*n* = 53)	Remimazolam (*n* = 57)
Tonsillectomy	15 (28.3)	13 (22.8)
Adenoidectomy	18 (34.0)	17 (29.8)
Combined tonsillectomy and adenoidectomy	20 (37.7)	27 (47.4)

Values are presented as numbers (%).

**Table 3:** Comparison of FLACC scores at extubation.

FLACC Category at Extubation	Control (*n* = 53)	Remimazolam (*n* = 57)	*p*-Value
FLACC = 0	32 (60.4)	45 (78.9)	0.047 *
FLACC 1–3	15 (28.3)	11 (19.3)	
FLACC ≥ 4	6 (11.3)	1 (1.8)	

Values are presented as numbers (%). FLACC: Face, Legs, Activity, Cry, and Consolability. * Fisher–Freeman–Halton exact test.

**Table 4:** Comparison of complications and safety.

Variable	Control (*n* = 53)	Remimazolam (*n* = 57)	*p*-Value
Postoperative vomiting at 5 min after extubation	4 (7.5)	1 (1.8)	0.194 *
Laryngospasm after extubation	0 (0)	0 (0)	—
Bronchospasm after extubation	3 (5.7)	0 (0)	0.109 *

Values are presented as numbers (%). * Fisher’s exact test. No *p*-value was calculated for laryngospasm because no events occurred in either group.

## Data Availability

The data presented in this study are available on request from the corresponding author. The data are not publicly available due to privacy restrictions and ethical considerations.

## Supplementary Materials

The following supporting information can be downloaded at https://www.mdpi.com/article/10.3390/biomedicines14092115/s1: Table S1. Longitudinal PAED and FLACC scores during the first 30 min after extubation.

## Abbreviations

The following abbreviations are used in this manuscript:

ASA	American Society of Anesthesiologists
CI	confidence interval
EA	emergence agitation
ED	emergence delirium
FLACC	Face, Legs, Activity, Cry, and Consolability
PACU	post-anesthesia care unit
PAED	Pediatric Anesthesia Emergence Delirium
POV	postoperative vomiting
