# Supplementary material for: End-of-Surgery Remimazolam Bolus Reduces Emergence Delirium in Children Undergoing Tonsillectomy and Adenoidectomy: A Randomized, Triple-Blind, Placebo-Controlled Trial
Source: Biomedicines. 2026 Sep 19;14(9):2115. doi: 10.3390/biomedicines14092115 (PMC13604081; doi:10.3390/biomedicines14092115)
Supplement: Supplementary file 1 [file biomedicines-14-02115-s001.zip › biomedicines-4499697-supplementary.pdf]

Supplementary Table S1. Longitudinal PAED and FLACC scores during the first 30 min after extubation

Outcome	Time point	Remimazolam (n=57)	Control (n=53)
PAED score	Immediately after extubation	4 [3–5]	5 [5–8]
	10 min	6 [5–7]	7 [6–8]
	20 min	7 [6–8]	8 [7–9]
	30 min	8 [7–9]	9 [8–9]
FLACC score	Immediately after extubation	0 [0–0]	0 [0–2]
	10 min	0 [0–0]	0 [0–2]
	20 min	0 [0–0]	0 [0–1]
	30 min	0 [0–0]	0 [0–1]

Values are presented as median [interquartile range]. PAED, Pediatric Anesthesia Emergence Delirium; FLACC, Face, Legs, Activity, Cry, and Consolability.
